# Evaluating Temperature Sensitivity of Vesicular Stomatitis Virus–Based Vaccines

**DOI:** 10.3201/eid2508.190281

**Published:** 2019-08

**Authors:** Derek R. Stein, Patrycja Sroga, Bryce M. Warner, Yvon Deschambault, Guillaume Poliquin, David Safronetz

**Affiliations:** Public Health Agency of Canada, Winnipeg, Manitoba, Canada (D.R. Stein, Y. Deschambault, G. Poliquin, D. Safronetz);; University of Manitoba, Winnipeg (P. Sroga, B.M. Warner, G. Poliquin, D. Safronetz)

**Keywords:** Viral hemorrhagic fever, Ebola virus, Lassa virus, vaccine stability, vaccines, viruses, Canada, vesicular stomatitis virus, temperature stability

## Abstract

Use of the vesicular stomatitis virus (VSV)–based Ebola virus vaccine during outbreaks and the potential use of a similar VSV-based Lassa virus vaccine has raised questions about the vaccines’ stability should the cold chain fail. We demonstrated that current cold chain conditions might tolerate significant variances without affecting efficacy.

Ebola virus (EBOV; family *Filoviridae*, genus *Ebolavirus*) and Lassa virus (LASV; family *Arenaviridae*, genus *Mammarenavirus*) are prominent etiologic agents of viral hemorrhagic fever diseases in humans that have variable but typically high death rates (*1*,*2*). At least 20 major EBOV outbreaks have occurred in sub-Saharan Africa, including 2 during 2018–2019. During 2013–2016, the largest documented EBOV outbreak caused ≈28,000 cases and ≈11,000 deaths in several countries in West Africa (*1*). In response to the extent of the outbreak, the development of experimental vaccines was accelerated, culminating in a ring vaccination trial of the live-attenuated vesicular stomatitis virus–based EBOV vaccine (VSVΔG/EBOVGPC) (*3*). Based on the success of the trial, a similar strategy is being used in the 2018–2019 outbreak in the Democratic Republic of the Congo; preliminary results from the World Health Organization have identified the vaccine as >97% effective (*4*).

LASV, a rodentborne virus, is endemic to most of West Africa. Annually, ≈300,000–500,000 LASV infections occur. Most are acquired after direct contact with infected rodents or their contaminated excreta or secreta (*5*). Several large LASV outbreaks have occurred, most recently in Nigeria. During 2015–2018, prolonged and severe Lassa fever outbreaks were documented across most of the country (*6*).

VSVΔG/EBOVGPC and a similar VSV-based LASV vaccine, VSVΔG/LASVGPC, are leading candidates to help reduce illnesses and death from EBOV and LASV infections (*7*). Under manufacturer recommendations, these products are intended to be maintained at –80°C. Given the often remote locations where EBOV and LASV emerge, concern exists about maintaining such a rigorous cold chain to deliver the vaccines to areas where they are most needed. We evaluated the temperature sensitivity of VSVΔG/EBOVGPC and VSVΔG/LASVGPC in vitro and their ability to provide protection against lethal LASV infection.

## The Study

To evaluate the effects of prolonged and multiple breaks in the cold chain on titer and protective efficacy of VSVΔG/EBOVGPC and VSVΔG/LASVGPC, we thawed cryopreserved stock vials of experimental-grade VSVΔG/EBOVGPC and VSVΔG/LASVGPC and maintained them for 7 d at 4°C, room temperature (≈21°C), or 32°C. In addition, we freeze–thawed (–80°C to room temperature) a vial of each stock 3 times over 7 days (3× freeze–thaw group). After incubation, we diluted the test vaccines according to the original stock titer and vaccinated groups of 9 outbred female Hartley guinea pigs (350–400 g) by the intraperitoneal route. A control group (9 animals per vaccine) comprising vaccination with 1 × 10^6^ PFU of stock unmanipulated VSVΔG/EBOVGPC or VSVΔG/LASVGPC was included with each condition. A mock vaccination control group (9 animals per vaccine) received the equivalent dose of an irrelevant VSV-based Andes virus vaccine (VSVΔG/ANDVGPC). For each group of vaccinated animals, we randomly selected 6 for monitoring survival and 3 for timed necropsy and analysis when control animals demonstrated advanced signs of disease. All animal work was approved by the Canadian Science Centre for Human and Animal Health’s Institutional Animal Care and Use Committee and was conducted according the guidelines of the Canadian Council on Animal Care. All work with infectious materials was conducted in the National Microbiology Laboratory’s Biosafety Level 4 laboratory. After vaccination, the test and control vaccines were titered on Vero E6 cells using 10-fold serial dilutions and standard plaque assay methods.

Twenty-eight days after vaccination, we challenged the guinea pigs with 1,000 times the median lethal dose (LD_50_; equivalent to 22 PFU) of guinea pig–adapted (GPA) EBOV, or 10 × LD_50_ (equivalent to 1 × 10^4^ 50% tissue culture infectious dose [TCID_50_]) of GPA-LASV. After challenge, we monitored animals daily, recording body weights and monitoring temperatures using previously implanted IPTT-300 temperature transponders using a DAS 6002 hand-held scanner (Bio Medic Data Systems, https://www.bmds.com).

**Figure 1:**
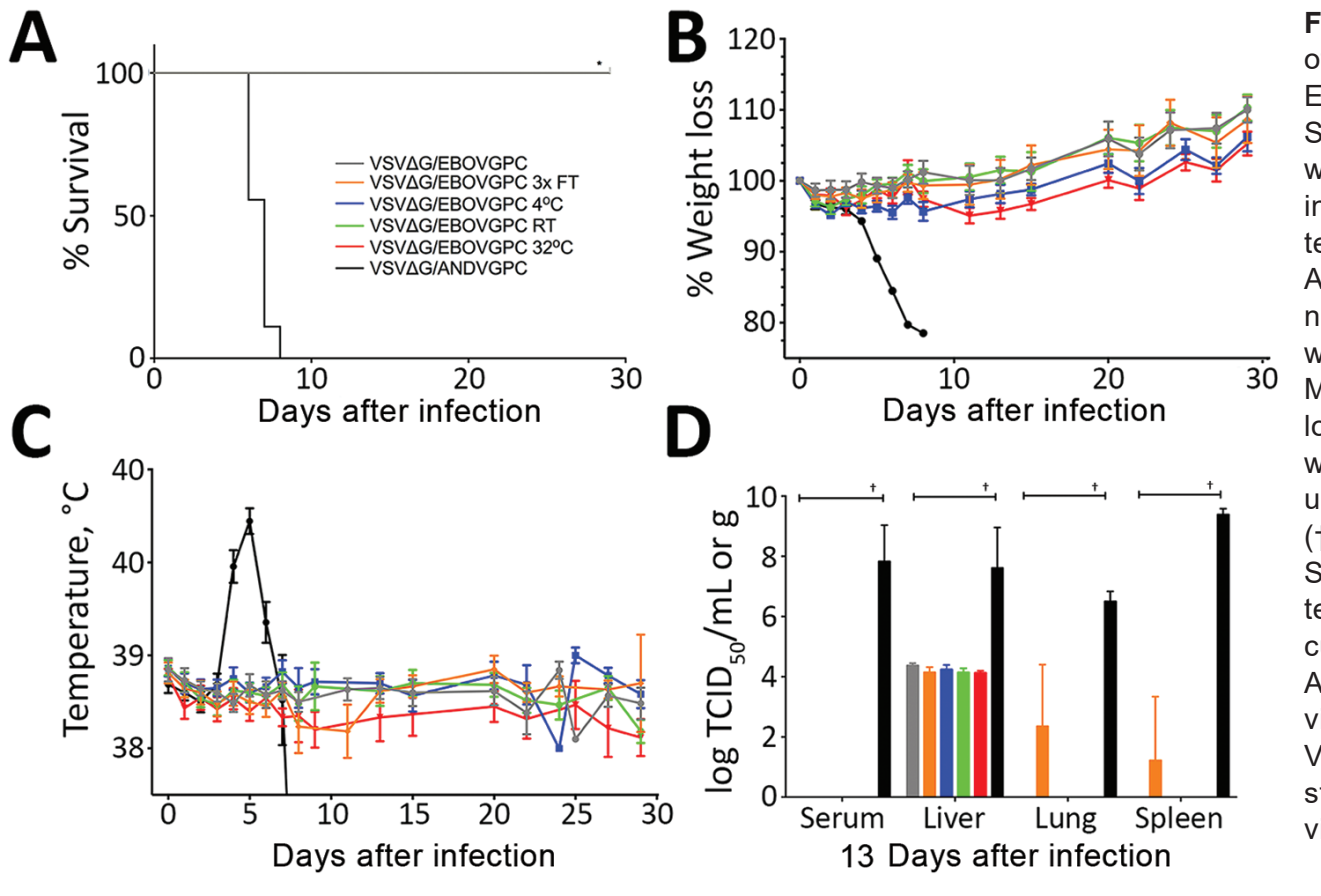
Evaluation of the effects of suboptimal storage of VSVΔG/EBOVGPC in guinea pigs. A) Survival rates. B) Percentage weight loss. Values >100% indicate weight gain. C) Body temperatures. D) Viral titers. In A, B, and C, n = 6 animals; in D, n = 3 animals. Survival analysis was conducted using a log-rank Mantel-Cox test (*p<0.0001). Viral loads in tissues were compared with VSVΔG/ANDVGPC controls using a 2-way analysis of variance (†p<0.0001). Error bars indicate SEM. FT, freeze–thaw; RT, room temperature; TCID_50_, 50% tissue culture infectious dose; VSVΔG/ANDVGPC, vesicular stomatitis virus–based Andes virus vaccine; VSVΔG/EBOVGPC, vesicular stomatitis virus–based Ebola virus vaccine.

The in vitro titer resulting from vaccination with VSVΔG/EBOVGPC was relatively stable across all conditions; only the 32°C test group showed a significant decrease (Table). This finding did not affect vaccine-derived protection from a GPA-EBOV challenge; we observed 100% survival across all conditions (Figure 1, panel A). After challenge, all vaccinated animals demonstrated no weight loss or increased body temperature (Figure 1, panels B, C). In contrast, sham-vaccinated animals (VSVΔG/ANDVGPC) demonstrated increased temperatures and decreases in body weights before reaching the study humane endpoints. Once the control animals achieved the humane endpoint, 3 animals per test group were euthanized to compare viremia and tissue titers. Vaccinated animals demonstrated significantly lower, and in many cases no, detectable infectious EBOV in tissue and blood samples (Figure 1, panel D).

**Table:** Comparison of in vitro titers after 7 days of suboptimal storage of vesicular stomatitis–based vaccines*

Vaccine	Condition	Actual dose, PFU	Fold change	% Survival
VSVΔG/EBOVGPC	Stock vaccine	4.69 × 10^6^	†	100
	3× freeze–thaw	1.97 × 10^6^	2.38	100
	4°C for 7 d	4.28 × 10^6^	1.10	100
	Room temperature for 7 d	9.11 × 10^5^	5.15	100
	32°C for 7 d	2.31 × 10^4^	203.00	100

VSVΔG/LASVGPC	Stock vaccine	1.29 × 10^6^	†	83
	3× freeze–thaw	7.43 × 10^5^	1.74	100
	4°C for 7 d	1.05 × 10^6^	1.23	83
	Room temperature for 7 d	2.86 × 10^5^	4.51	83
	32°C for 7 d	3.75 × 10^4^	34.40	66

*VSVΔG/EBOVGPC, vesicular stomatitis virus–based Ebola virus vaccine; VSVΔG/LASVGPC, vesicular stomatitis virus–based Lassa virus vaccine. †Stock vaccine was not subject to any treatments and thus had no -fold change. The fold change for all other treatment groups was calculated on the basis of the original dose of stock vaccine.

**Figure 2:**
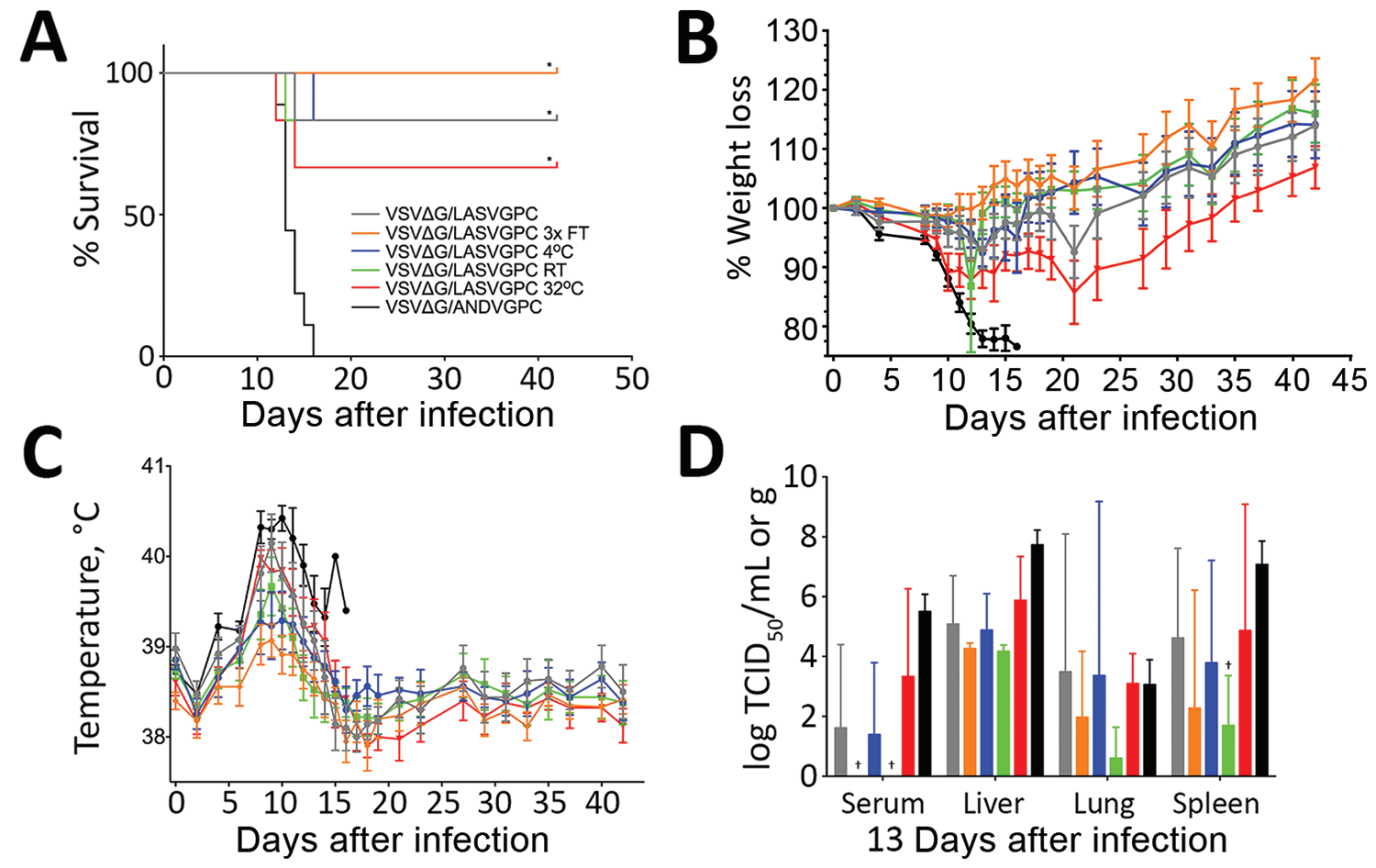
Evaluation of the effects of suboptimal storage of VSVΔG/LASVGPC in guinea pigs. A) Survival rates. B) Percentage weight loss. Values >100% indicate weight gain. C) Body temperatures. D) Viral titers. For A, B, and C, n = 6; for D, n = 3. Survival analysis was conducted using a log-rank Mantel-cox test. Viral loads in tissues were compared with VSVΔG/ANVDVGPC controls using a 2-way analysis of variance. *p<0.0001; †p = 0.002. Error bars indicate SEM.FT, freeze–thaw; RT, room temperature; TCID_50_, 50% tissue culture infectious dose; VSVΔG/ANDVGPC, vesicular stomatitis virus–based Andes virus vaccine; VSVΔG/LASVGPC, vesicular stomatitis virus–based Lassa virus vaccine.

VSVΔG/LASVGPC was similarly stable across all experimental groups but to a lesser degree in the 32°C group than in VSVΔG/EBOVGPC (Table). Despite the relative stability, deaths occurred in most groups, including the positive control vaccinated animals (Figure 2, panel A). The exception was in the 3× freeze–thaw group, which maintained 100% survival. Consistent with these findings, we noted weight loss and increased body temperatures in all but the 3× freeze–thaw groups (Figure 2, panels B, C). Sham-vaccinated animals progressed as previously described after challenge, and infection was uniformly lethal within 18 days after challenge. Similarly, we noted infectious virus at varying levels in some or all samples tested in all groups (Figure 2, panel D).

## Conclusions

VSVΔG/EBOVGPC was surprisingly durable despite 7 days of suboptimal storage with no obvious clinical signs of disease and no deaths among animals across the treatment groups. We detected low-level viral titers in liver samples collected when the control animals were experiencing terminal disease. The 3× freeze–thaw condition resulted in detectable virus in both lung and spleen, although 100% of animals still survived. Curiously, VSVΔG/LASVGPC demonstrated improved performance under the same condition; however, a small sample size precludes any definitive conclusion.

The other temperature conditions had little effect on VSVΔG/LASVGPC in vitro titers. However, in most animals we noted clinical signs of disease, including lethargy, inappetence, moderate to severe increases in body temperature, and mild to moderate decreases in body weight. Consistent with these findings, experimental animals sampled when control animals were perimortem had infectious viral titers that were not significantly different from those of the control animals. Nevertheless, statistically significant increases in survival rates occurred in all experimental groups. Although 32°C proved more deleterious than other temperature conditions, the efficacy was similar to the control vaccination group. The GPA-LASV model is relatively new and has been adapted for maximum lethality in Hartley guinea pigs. VSVΔG/LASVGPC has proved to be extremely effective in both nonhuman primates and strain 13 guinea pigs when tested against several genetically and geographically distinct wild-type LASVs (*8*). This slight reduction in efficacy has been noted previously and might speak to the aggressiveness of the outbred guinea pig Lassa fever model (*9*). Follow-up studies in the inbred strain 13 guinea pig model, which is better characterized, could be considered (*10*).

Every effort needs to be made to ensure optimal storage and dosages of medical countermeasures to treat and prevent human disease. However, in remote and often tropical areas, maintaining these standards can be challenging, particularly if, in addition to climatic conditions, civil conflict is ongoing. Vaccine shortages are problematic, particularly during outbreaks, as demonstrated by the recent yellow fever vaccine shortage (*11*). Enhanced knowledge of vaccine stability under suboptimal storage conditions can help mitigate shortage issues by ensuring breaks in the cold chain do not necessarily translate into unusable vaccine lots. Our data demonstrate that the current –80°C cold chain condition might tolerate significant variances without affecting efficacy, at least in animals. 
